# Gut microbiota-metabolome crosstalk in allergic diseases: mechanistic insights and translational opportunities

**DOI:** 10.3389/falgy.2025.1631479

**Published:** 2025-07-15

**Authors:** HanBin Qin, Jiaxin Sui, Shuang Wang, Xiaojing Lv, Zile Zhang, Xinhua Lin, Xuexia Liu, Hua Zhang

**Affiliations:** 1 School of Clinical Medicine, Shandong Second Medical University, Weifang, China; 2 Department of Otorhinolaryngology, Head and Neck Surgery, Yantai Yuhuangding Hospital, Qingdao University, Yantai, China; 3 Shandong Provincial Key Laboratory of Neuroimmune Interaction and Regulation, Yantai, China; 4 Shandong Provincial Clinical Research Center for Otorhinolaryngologic Diseases, Yantai, China; 5 Yantai Key Laboratory of Otorhinolaryngologic Diseases, Yantai, China; 6 Qingdao Medical College of Qingdao University, Qingdao, China; 7 The 2nd Medical College of Binzhou Medical University, Yantai, China; 8 Shandong Stem Cell Engineering Technology Research Center, Central Laboratory, Affiliated Yantai Yuhuangding Hospital of Qingdao University, Yantai, China

**Keywords:** allergic diseases, gut microbiota, metabolome, microbiota dysbiosis, mechanisms of immunity

## Abstract

The gut microbiota and its metabolites play important roles in the pathogenesis of various diseases. The diversity of the gut microbiota is closely related to the development and function of the human immune system. Dysbiosis, characterized by alterations in the species, quantity, and distribution of microbial community, may disrupt immune tolerance mechanisms, thereby inducing excessive immune responses to allergens and increasing the risk of allergic diseases. Various metabolites, such as short-chain fatty acids (SCFAs), bile acids, and amino acid metabolites, exert significant regulatory effects on the development of allergic diseases by modulating immune cell function, maintaining intestinal barrier integrity, and participating in signal transduction pathways. A comprehensive investigation into the relationship between allergic diseases and gut microbiota and their metabolites not only aids in elucidating the pathogenesis of allergic diseases but also provides novel insights and a theoretical foundation for developing innovative diagnostic methods, preventive strategies, and therapeutic options. This article systematically reviews the latest findings regarding the mutual influence between gut microbiota and the metabolome in host immune regulation, as well as the impact of this interaction on the development of allergic diseases, aiming to offering new strategies for the prevention and treatment of allergic diseases.

## 1 Introduction

Human microbiome refers to the microbial communities residing in various parts of the human body, including bacteria, fungi, viruses, and archaea. A wide range of anatomical sites, including the skin, mucous membranes, respiratory tract, uterus, vagina, and digestive tract, harbor complex microbial ecosystems that are specifically adapted to the unique characteristics of each niche (1, 2). These microorganisms engage in dynamic interaction with the human host, forming a highly complex ecosystem that profoundly influences health and disease states (3). The skin, being the largest human organ with a surface area of approximately 2 m^2^, exhibits higher microbial community composition in areas rich in sebum and moisture (4–6). Compared with the lower gastrointestinal tract and skin, the respiratory tract demonstrates a lower microbial density. Furthermore, microbial density varies along the respiratory tract, with the upper respiratory tract exhibiting greater density and diversity compared to the lower respiratory tract (7, 8). The microbial ecosystem within the digestive tract is the most complex, diverse, and abundant across the entire body (9). The majority of these microorganisms are found in the oral mucosa and gastrointestinal tract, where the gut microbiota plays an important role in nutrition metabolism, immune regulation, and disease prevention (10). The gut microbiota contains approximately 10^9 to 10^11 microorganisms per gram of luminal content, with species diversity ranging from 1,000 and 3,000 (2, 3, 11). One gram of human feces can harbor 10^10 to 10^11 bacteria. The human gut microbiota is primarily composed of *Firmicutes, Bacteroidetes, Actinobacteria,* and *Proteobacteria* (12, 13). More than 1,000 bacteria species reside in the intestines, and their metabolites can generally be categorized into the following categories: SCFAs (such as acetate, propionate, butyrate), long-chain fatty acids (LCFAs), bile acids (primary bile acids and secondary bile acids), amino acid metabolites (e.g., ammonia, amine, indole and its derivatives),vitamins, and other metabolites such as trimethy lamine and its oxidants form (TMAO), hydrogen, methane, and carbon dioxide (14, 15) (Table 1). In certain studies, the gut microbiota has been redefined as an “important organ,” and the gut microbiota along with its metabolome have been demonstrated to influence the regulation, maturation, and function of the immune system, playing a crucial role in the differentiation of immune cells (16, 17). Many animal model studies have demonstrated that alterations in intestinal bacterial communities can influence the onset of diseases in distant organs, thereby giving rise to the concepts of the lung-gut axis and the skin-gut axis (18–20). SCFAs, such as propionic acid, butyric acid, and acetate, are the end products of dietary fiber fermentation by the gut microbiota. These metabolites can reach distant organs and exert beneficial effects on the immune system. For instance, acetate, produced by members of the *Lachnospiraceae* family within the gut, has been shown to activate the innate immune system in the lungs and subsequently modulate the skin's immune defense mechanism (21, 22).

**Table 1:** Main classifications and sources of the metabolome of the gut microbiota and their main role in allergic diseases.

Classification of gut microbial metabolites	Base	Source	Main functions in allergic diseases
Short-chain fatty acid	Acetic acid, propionic acid, butyric acid	Produced by intestinal bacteria (e.g., Mycobacterium avium and Mycobacterium thickum) through fermentation of dietary fiber and resistant starch	Energy supply: butyric acid is the main energy source for colonic epithelial cells, providing 60–70% of energy requirements. Anti-inflammatory effects: Inhibits the production of pro-inflammatory cytokines and promotes the secretion of anti-inflammatory cytokines (e.g., IL-10) through the activation of G protein-coupled receptors (e.g., GPCR43 and GPCR109A). Regulate intestinal barrier function: enhance the expression of tight junction proteins and reduce intestinal permeability. Regulates immune cell function: promotes the differentiation of regulatory T cells (Tregs) and inhibits the differentiation of Th1 and Th17 cells.
Long chain fatty acid	Saturated fatty acids, monounsaturated fatty acids, and polyunsaturated fatty acids	Obtained primarily from food and, to a lesser extent, through synthesis of long-chain fatty acids in the liver and other tissues from ingested carbohydrates and proteins	Involved in cell signaling: arachidonic acid generates inflammatory mediators such as prostaglandins and leukotrienes through the cyclooxygenase (COX) and lipoxygenase (LOX) pathways, which play a key role in immune responses and inflammatory processes. The metabolites of the polyunsaturated fatty acids EPA and DHA (e.g., lipid mediators) have anti-inflammatory effects and are involved in immune regulation and tissue repair. Anti-inflammatory effects: Polyunsaturated fatty acids (e.g., EPA and DHA) exert anti-inflammatory effects by inhibiting the NF-*κ*B signaling pathway and reducing the production of pro-inflammatory cytokines (e.g., TNF-α, IL-6, IL-1β). Regulation of immune cell function: Long-chain fatty acids regulate the activation and function of immune cells by affecting the fluidity of cell membranes.
Bile acids	Bile acids, goose deoxycholic acid and its derivatives	Bile acids are synthesized primarily by the liver and converted by gut microbiota	Metabolism regulation: regulates energy metabolism, glucose metabolism and lipid metabolism through activation of farnesylate X receptor (FXR) and G protein-coupled bile acid receptor (TGR5). Immunomodulation: regulates immune cell function and reduces inflammatory response.
Amino acid metabolite	Tryptophan metabolites Indole, indoleacetic acid, indole-3-acetaldehyde	Produced by gut microbes by breaking down tryptophan	Regulating immune response: regulates immune cell function and reduces inflammatory response by activating AhR (Aromatic Hydrocarbon Receptor). Regulate intestinal barrier function: Enhance the tight junction of intestinal epithelial cells and reduce intestinal permeability.
	Polyamine	Produced by gut microbes through the breakdown of amino acids (e.g., ornithine, lysine)	Cell proliferation and differentiation: polyamines are important regulators of cell proliferation and differentiation. Anti-inflammatory effect: reduce inflammatory response by regulating immune cell function. Regulation of intestinal barrier function: enhance the tight junctions of intestinal epithelial cells and reduce intestinal permeability.
	Nitric oxide	Gut microbes produce arginine by breaking down	
Vitamins	Water-soluble vitamins B-complex and C, fat-soluble vitamins A, D, E, K	Vitamin K and some of the B-complex vitamins, through dietary intake, need to be synthesized by some bacteria	Promote the growth of beneficial bacteria Modulates the immune response

**Figure 1:**
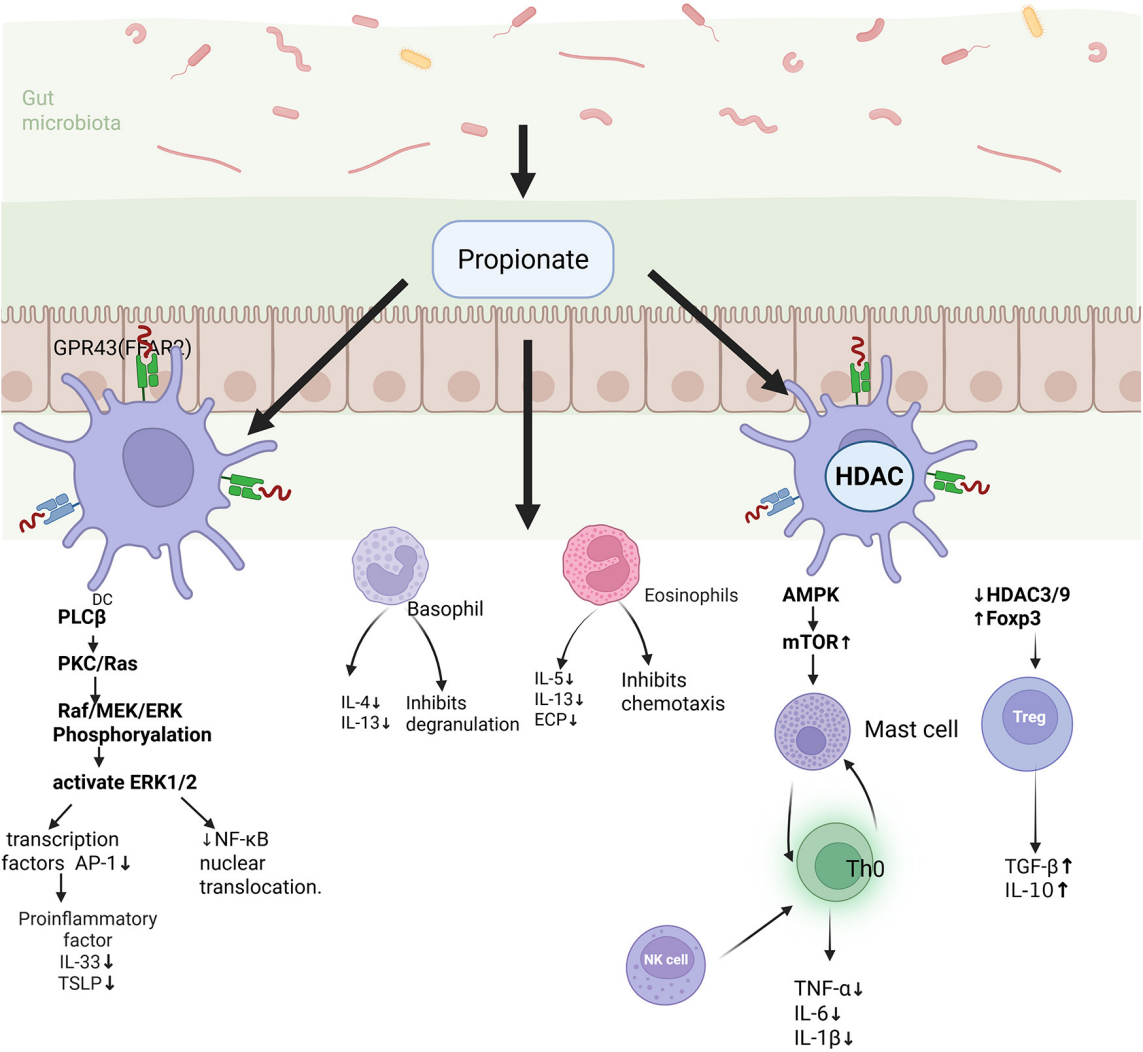
Immune response of the body after exposure to allergens. Immune response following allergen exposure in the body. Upon entry through the intestinal epithelium, allergens are captured by dendritic cells (DCs) and presented to naïve T cell expressing IL-4 (Th0), which subsequently differentiate into Th2 cells. Th2 cells secrete cytokines such as IL-4, IL-5, and IL-13, promoting B cell production of IgE antibodies while also recruiting eosinophils and basophils to the site of inflammation. IgE antibodies blind to receptors on mast cells and basophils, upon re-exposure to the same allergen, these cells become activated, releasing inflammatory mediators including histamine and thereby inducing allergic symptoms. Simultaneously, the gut microbiota and mucus layer play critical roles in modulating immune responses and preventing allergen penetration.

Allergic diseases, also referred to as hypersensitivity disorders, can manifest at various stages of life, from infancy to old age. These conditions involve an abnormal immune response of the body to typically harmless substances, known as allergens (23). The pathogenesis of allergic diseases primarily involves an exaggerated immune reaction, particularly IgE-mediated responses (Figure 1). Globally, allergic diseases affect approximately 15% of the population and have shown a significant increase in incidence in recent years, with industrialized countries experiencing a marked rise (24). According to the latest report by the World Health Organization's (WHO), asthma is currently one of the most prevalent chronic diseases worldwide, affecting approximately 339 million individuals. Since the 1980s, the incidence rate of asthma has significantly increased, especially among children and in urban areas (25). Additionally, the prevalence of allergic rhinitis (AR) has risen by 50% over the past three decades, while food allergies has doubled globally. The high incidence of these diseases not only severely impacts patients’ quality of life but also imposes a substantial economic burden on public health system. Studies indicate that the treatment costs for allergic diseases account for more than 2% of global healthcare expenditures (26). With the continued rise in incidence rate, it is anticipated that the proportion of allergic diseases treatment costs in global healthcare expenditure may further increase in the coming years.

Research demonstrates that patients with allergic diseases often exhibit dysbiosis of their microbiota. In patients with skin bacteria, the diversity of skin bacteria is significantly reduced, accompanied by an increase in *Staphylococcus aureus*, and a decrease in beneficial bacteria such as *Staphylococcus epidermidis* (20, 27–29). Similarly, individuals with allergic diseases exhibit significantly lower gut bacterial diversity compared to healthy controls, characterized by a reduction in beneficial bacteria like *Lactobacillus* and an overgrowth of potentially harmful bacteria such as *Escherichia coli*. This dysbiosis of the microbiota is closely linked to the pathogenesis and progression of allergic diseases (30, 31). Beneficial bacteria play a crucial role in regulating the development and function of the immune system, promoting the differentiation of regulatory T cells (Tregs), inhibiting T helper 2 cell (Th2)-type immune responses, and thereby reducing IgE production and allergic reactions (32, 33). This microbial imbalance may lead to excessive immune responses and elevate the risk of allergic diseases (34). Metabolites produced by the gut microbiota influence immune responses by modulating the activity of immune cells. Acting as signaling molecules, these microbiota metabolites activate or inhibit intracellular signaling pathways, regulate gene expression, and modulate cell functions (35). Furthermore, by enhancing the barrier function of intestinal epithelial cells, these metabolites prevent allergens from entering the bloodstream, thereby mitigating systemic allergic reactions (Table 2).

**Table 2:** The relationship between gut microbiota metabolites and early and late stages of allergic diseases, as well as immune mechanisms.

Classification of gut microbial metabolites	Mechanism of action in the early stage of disease (sensitization stage)	Late-stage disease (chronic phase) action mechanism
Short-chain fatty acid	Immune tolerance induction: Butyric acid promotes Treg differentiation by inhibiting HDAC (increasing Foxp3 and IL-10) and inhibits Th2 polarization (decreasing IL-4 and IL-13). Propionic acid inhibits DC release of TSLP/IL-33 via GPR43.	Barrier repair obstacle: SCFAs reduction leads to tight junction protein (occludin↓) destruction, worsening allergen penetration. Insufficient histone acetylation promotes activation of fibrosis genes (TGF-β↑).
Long chain fatty acid	*ω*-3 anti-inflammatory: Resolvins derived from DHA/EPA inhibit degranulation of mast cells (↓ histamine). ω-6 pro-allergic: Arachidonic acid (AA) promotes release of PGD2/LTC4, recruiting eosinophils.	Chronic inflammation:- Imbalance of *ω*-6/ω-3 ratio → sustained Th2 response (IL-5↑, IgE↑)- Lipid dysregulation leads to fibrosis of airway/skin.
Bile acids	Immune regulation: Primary bile acids (CA) inhibit mast cell activation through FXR (Fc*ε*RI signal↓). Regulate intestinal flora, reduce allergenic bacteria (such as Clostridia).	Disorder of bile acid metabolism: Secondary bile acids (DCA) activate keratinocytes (IL-8↑) → skin barrier damage. Liver-intestine circulation disorders exacerbate systemic allergies.
Amino acid metabolite	Indole protection: Tryptophan → indole derivatives (AhR ligands) promote IL-22 secretion, enhance epithelial barrier. Histamine explosion: Mast cell degranulation → increased vascular permeability,acute allergic symptoms.	Chronic injury: Prolonged release of histamine → H1R desensitization, decreased therapeutic effect. Depletion of spermine → autophagy defect → impediment in clearing apoptotic cells (chronic eczema).
Vitamins	Vit D3: Inhibit Th2 polarization (↓GATA3) and promote antimicrobial peptides (LL-37) to repair skin. Vit B2: Support symbiotic bacteria (such as F. prausnitzii) to produce SCFAs.	Vitamin D deficiency: Abnormal differentiation of corneal cells (decreased filaggrin) → permanent damage to the barrier. Vitamin B6 deficiency → tryptophan metabolism tends towards indolamine (pro-inflammatory).

The alterations in the composition and function of the gut microbiota can influence the development and function of the immune system, thereby contributing to the onset of allergic diseases. Metabolomics not only serves as a valuable tool for identifying biological markers but also plays a critical role in disease diagnosis and treatment monitoring. By integrating data on intestinal microbial communities with metabolomics analysis, we can gain deeper insights into the overall functional impact of the gut microbiota on host health, as well as the effects of host-derived metabolomics changes on resident bacterial populations (36). This approach facilitates the development of more effective prevention and treatment strategies for allergic diseases.

## 2 SCFAs and allergic diseases

SCFAs are defined as fatty acids with a carbon chain length of 1–6 carbon atoms, including acetate, propionate, and butyrate. In the intestine, SCFAs are primarily produced through the fermentation of dietary fiber and resistant starch by the gut microbiota, particularly involving bacterial genera such as *Bacteroides* and *Akkermansia* (37, 38). Changes in the quantity, species, or proportions of these bacterial populations within the gut can lead to corresponding alterations in the production and composition of SCFAs (12). Among these, butyrate is considered one of the most important SCFAs due to its critical physiological roles (12, 39).

As an essential energy source for colonocytes, SCFAs play a critical role in maintaining intestinal health. Butyrate, specially, generates ATP in cellular mitochondria via the β-oxidation pathway, supplying approximately 70% of the energy requirements for colonocytes. During the development of AR, the population of beneficial bacteria such as *Lactobacillus* and *Bifidobacterium* decrease, potentially leading to reduced production of SCFAs, particularly acetate. Simultaneously, certain harmful bacteria of the *Clostridium genus* may over proliferate, altering the levels and ratios of SCFAs, including butyrate. The reduction in butyrate can disrupt intestinal cell energy metabolism, inducing apoptosis and impairing intestinal barrier function. This dysfunction allows inflammatory factors to enter the bloodstream, triggering systemic allergic reactions (40).

**Figure 2:**
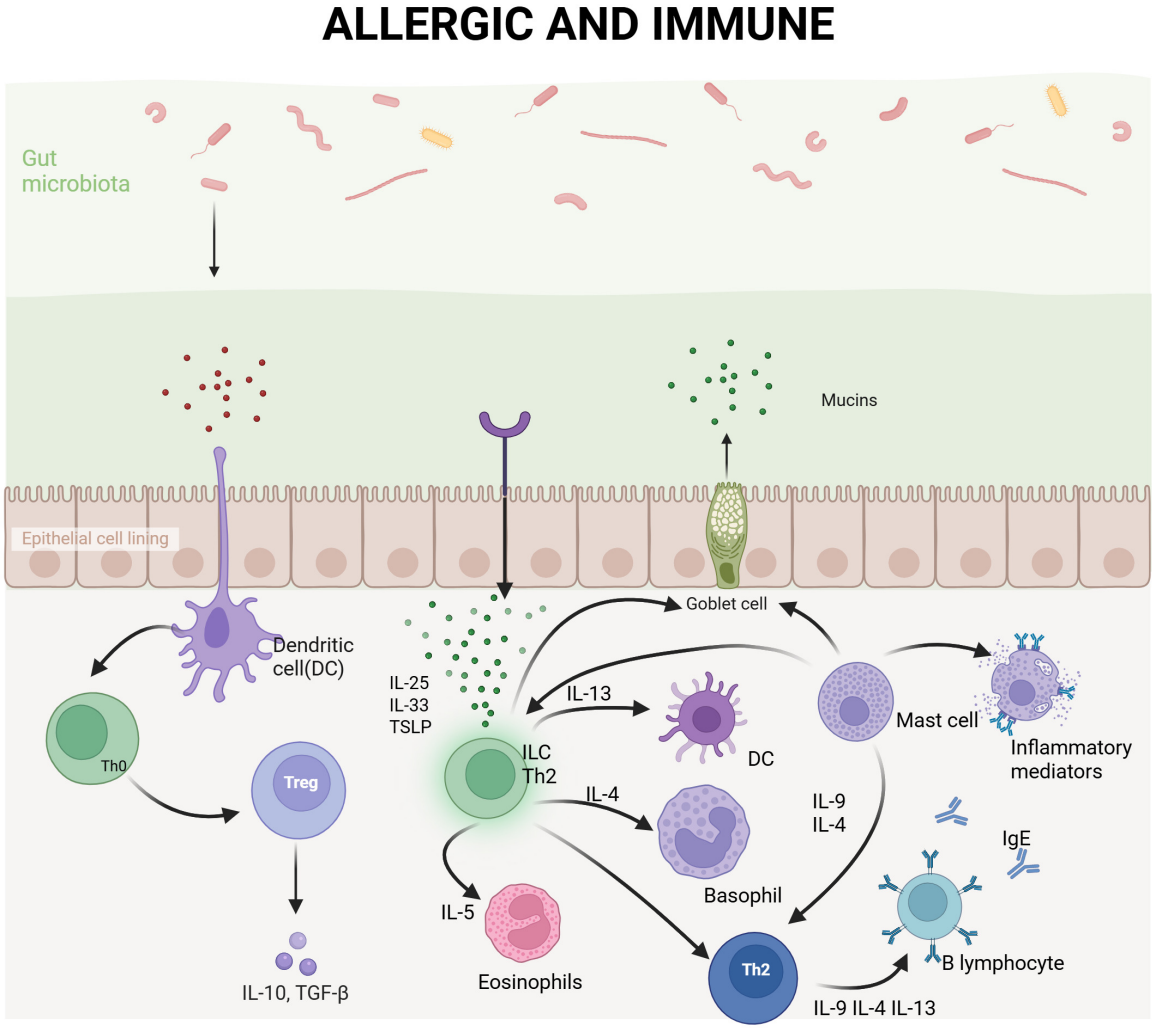
Major mechanisms by which SCFAs modulate allergic airway disease. SCFAs are produced via the fermentation of dietary fibers by the gut microbiota and exert anti-inflammatory effects in allergic diseases through the activation of GPR41/GPR43 receptors on immune cells. SCFAs suppress DCs from releasing pro-inflammatory cytokines such as IL-33 and TSLP, thereby attenuating Th2 immune responses. Simultaneously, they promote the differentiation of Tregs and enhance IL-10 secretion, contributing to immune tolerance. Additionally, SCFAs downregulate pro-inflammatory mediators, including TNF-α and IL-6, in macrophages, inhibit eosinophil degranulation and basophil chemetaxis, and reduce levels of Th2-associated cytokines (IL-5, IL-13) and eosinophil cationic protein (ECP), thus comprehensively mitigating allergic inflammation.

SFCAs not only serves as an essential energy source for intestinal cells but also play a pivotal role in regulating immune cells function (41) (Figure 2). In allergic diseases, SCFAs function as naturally occurring immune modulators, contributing significantly to regulating the activity of immune cells, and acting as signaling molecules involved in various physiological processes. In Food allergies, propionate inhibits the NF-*κ*B pathway, thereby reducing the expression of pro-inflammatory cytokines such as TNF-α and IL-6 (42). Additionally, SCFAs can promote the differentiation of Tregs and enhance their secretion of IL-10, an important anti-inflammatory cytokine that suppresses the activation and proliferation of various immune cells, exerting potent anti-inflammatory effects (43). The hallmark of AR is a Th2-mediated immune response, characterized by elevated IgE levels and mast cells degranulation, which elicits allergic symptoms (44, 45). Tregs suppress the activation and proliferation of Th2 by secreting transforming growth factor-beta (TGF-β), thereby reducing the production of Th2 proinflammatory cytokines and inhibiting mast cells degranulation and the release of inflammatory mediators, thus alleviating the AR symptoms (46). Additionally, there exists a dynamic balance between Th17 and Treg. Th17 cells promote inflammatory cells infiltration and may induce changes in mucosal barrier function (40). A decrease in butyrate affects the proliferation and function of Tregs, increases the differentiation of Th17, disrupts immune homeostasis, and further exacerbates nasal inflammation and allergic reactions. Group 2 innate lymphoid cells (ILC2s) are increasingly recognized as key regulators of type 2 inflammation and are markedly elevated in human airway disease characterized by type 2 inflammatory, including AR and asthma (47). Gavin Lewis and his team demonstrated that propionate salts can significantly inhibit the production of type 2 cytokines by ILC2s *in vitro* and alleviate ILC2-dependent allergic inflammation, such as asthma, *in vivo*. Succinate salt have been shown to downregulate the expression of the critical transcription factor GATA-3, which governs ILC2 development and function (48). This reduction in GATA-3 expression decreases cellular metabolism, thereby regulating immune cell activity and inhibiting lung ILC2 function in asthma, as well as the subsequent development of airway hyperresponsiveness (AHR) (49). In allergic asthma, SCFAs enhances systematic delivery by upregulating intestinal monocarboxylic acid transporters (MCTs), thereby attenuating Th2/ILC2-mediated inflammatory responses and inhibiting eosinophil infiltration and lung mucin production (50–53). Furthermore, dendritic cells (DCs) serves as a central bridge between the innate and adaptive immune systems. In allergic diseases such as AR and allergic asthma, succinate and propionate salts can suppress T cell activation by inhibiting DC maturation, thus dampening the intensity of the immune response (54).

SCFAs function as agonists for several G protein-coupled receptors (GPCRs), thereby regulating immune modulation and intestinal barrier function (12, 38). GPCRs, including free fatty acid receptor 2 (FFAR2, or GPR43) and 3 (FFAR3 or GPR41), as well as hydroxycarboxylic acid receptor 2 (HCAR2 or GPR109A), are expressed in various cell types, such as intestinal epithelial cells and immune cells. SCFAs stimulate these GPCRs to activate mitogen-activated protein kinases (MAPKs) and extracellular signal-regulated kinases (ERK1/2), exerting immunoregulatory effects. Butyrate promotes cellular energy metabolism and maintains intestinal barrier integrity by activating GPCRs such as GPR43 and GPR109A (55). In AR, the activation of GPR43 and GPR41 by butyrate can inhibit the NF-*κ*B pathway, reduce pro-inflammatory cytokines production, and alleviate nasal mucosa inflammation.

SCFAs can also influence the occurrence and progression of allergic diseases by modulating the gut-lung axis. The gut-lung axis refers to the bidirectional communication pathway between the gut microbiota and the lungs (56). In an *in vivo* study, the Chinese herbal formula (Gu-Ben-Fang-Xiao Decoration, GBFXD), used for asthma treatment, was shown to alleviate lung inflammation while increasing the abundance of SCFAs-producing bacteria and SCFAs levels, especially acetate. SCFAs through binding to receptors such as GPR43, inhibit inflammation, regulate the differentiation and function of immune cells. SCFAs, particularly acetate, can enhance the differentiation and function of Tregs, thereby suppressing allergic airway inflammation. After treating mice with antibiotics, the protective effect of bone-resisting anti-soup on the lungs was weakened (57). These findings indicate that SCFAs derived from gut microbiota are important immunoregulators and contributors to the gut-lung axis. SCFAs derived from the gut microbiotaserve as critical immunoregulatory agents and play a pivotal role in the gut-lung axis.

Overall, multiple studies have demonstrated that the gut microbiotaproduce diverse SCFAs via fermentation, thereby exerting distinct anti-allergic effects. Additionally, the consumption of dietary fiber has been shown to modulate the composition of gut microbiota. Specifically, it reduces the abundance of the *Firmicutes* phylum while increasing that of *Bacteroidetes* at the phylum level, and enhances *Lactobacillaceae* at the family level. Therefore, supplementing the diet with foods rich in dietary fiber or directly administering SCFAs may serve as effective strategies for the treatment of allergic diseases. Future research should focus on elucidating the specific mechanisms by which SCFAs influence allergic diseases, developing personalized treatment regimens, and enhancing therapeutic efficacy.

## 3 LCFAs and allergic diseases

LCFAs in the gut generally refer to fatty acids with a carbon chain length of 12–20 carbon atoms, including saturated fatty acids, monounsaturated fatty acids, and polyunsaturated fatty acids (PUFAs). Among these, the PUFAs linoleic acid (n-6) and alpha-linolenic acid (n-3) play critical roles in human energy metabolism, cell membrane structure, and signal transduction (58). The n-6 long-chain polyunsaturated fatty acids (n-6 LCPUFA) primarily include arachidonic acid (AA), while the n-3 LCPUFA mainly consist of eicosapentaenoic acid (EPA) and docosahexaenoic acid (DHA). Dietary intake is the primarily source of LCFAs, although the human body can also synthesize certain LCFAs in the liver and other tissues from carbohydrates and proteins. Essential fatty acids, such as n-3 and n-6 PUFAs, must be obtained through diet.

LCFAs decrease the body's sensitivity to allergens by influencing the early development of the immune system (17). A prospective cohort study conducted in Sweden demonstrated that children who consumed higher levels of very long chain n-3 polyunsaturated fatty acids (n-3 VLCPUFAs) at the age of 8 exhibited a reduced risk of AR from ages 8 to 16. In this study, AA was identified as a mediator of inflammation due to its role as the precursor of pro-inflammatory eicosanoids, such as prostaglandin D2, which contribute to the establishment of an immune-allergic predisposition toward asthma. AA-derived prostaglandin E2 can suppress Th1 response while promoting Th2 responses, thereby influencing the balance of the body's immune response (59). A study by the University of Southampton in the UK indicated that during the early life development, breastfeeding promotes immune maturation, prevents infections, and may reduce the risk of allergies (26). AA and DHA are the primarily LCPUFAs found in human breast milk. The immune system of newborns undergoes development over several months to years, establishing balances between Th1 and Th2 responses, as well as a balance between effector T cells and Tregs (60–62). Impairments in immune development can lead to inadequate cellular responses or sustained immune imbalance (e.g., imbalance between Th1 and Th2 systems), increasing infants' susceptibility to allergens or predisposing them to immune-mediated diseases such as food allergies. Furthermore, studies have shown that supplementing pregnant women with n-3 LCPUFAs can modulate immune cell function and their responses in umbilical cord blood, typically characterized by inhibition of Th2-type responses, promotion of Th1 and Th2 balance, and enhancement of immune maturation (60, 63). Supplementation of pregnant women with n-3 LCPUFAs has been associated with reduced infants sensitivity to common food allergens, decreased risk and severity of atopic dermatitis during the first year of life, and reduced risk of persistent wheezing and asthma between the age of 3 and 5. Therefore, LCPUFAs play a critical role in the early prevention and management of allergic diseases.

LCFAs play a significant role in modulating the degree of inflammatory response in the body. In healthy individuals, the *Firmicutes*/*Bacteroidetes* (F/B) ratio is typically maintained within a relatively stable range. Studies have demonstrated that the F/B ratio is often decreased in patients with allergic diseases, reflecting an increased relative abundance of *Bacteroidetes* and a decreased relative abundance of *Firmicutes* (64–66). This alteration is closely associated with excessive immune response and inflammation. David Johane Machate reported that a high-fat diet enriched in LCFAs can increase the proportion of *Firmicutes and Proteobacteria* while decreasing the proportion of *Bacteroidetes*. Conversely, a diet rich in n-3-PUFAs can elevate the ratio of *Bacteroidetes* and *Actinobacteria* while reducing the ratio of *Firmicutes* and *Proteobacteria*, thereby mitigating inflammation. On the other hand, a diet abundant in n-6-PUFAs is linked to metabolic disorders such as obesity and inflammatory bowel disease (15).

The dietary intake of LCFAs may also influence the progression of allergic diseases. Research indicates that in the pathogenesis of allergic asthma, fatty acid binding protein 5 (FABP5) is regulated under IL-4/IL-13 stimulation, modulating the metabolism of LCPUFAs, particularly the accumulation of oleic acid. This process activates the PPARγ signaling pathway, promoting M2 macrophage polarization and exacerbating disease progression. Excessive intake of oleic acid may similarly worsen disease progression through this mechanism, suggesting a potential link between dietary fatty acids intake and the progression of asthma (67, 68).

LCFAs influence the balance of gut microbiotaand the progression of allergic diseases in the human body. Further investigation is required to elucidate the specific roles of LCFAs in allergic diseases and to explore their potential application in mitigating allergic reactions. Additionally, the significance of LCFAs in early immune system development and homeostasis, particularly regarding allergen sensitivity and allergic manifestations such as wheezing and asthma for LCPUFAs, warrants greater attention. Research suggests that LCPUFAs (typically a combination of EPA and DHA) confer immunomodulatory benefits during pregnancy, lactation, and infancy. However, the duration of these effects remains unclear. Therefore, clarifying the immunological and clinical impacts of LCPUFAs in infants and children, as well as the persistence of these effects, will provide valuable insights for future research directions.

## 4 Vitamins and allergic diseases

The human body requires a diverse of vitamins to maintain normal physiological functions and overall health. Vitamins are organic compounds, including water-soluble vitamins (the B complex and vitamin C) and fat-soluble vitamins (vitamins A, D, E, and K). Some vitamins cannot be synthesized by the human body or are produced in insufficient quantities, necessitating their acquistion through dietary intake. Notably, vitamins K and certain B vitamins (e.g., vitamin B12) must be synthesized by specific bacteria, such as *Lactobacillus*, which participate in the biosynthesis of vitamins B and K.

Vitamins modulate immune responses by altering the balance of the gut microbiota and its environment. Studies have demonstrated that certain vitamins can promote the growth and proliferation of beneficial bacteria, such as *Bifidobacteria* and *Akkermansia*. For instance, vitamin C exhibits antioxidant properties, reducing oxidative stress in the intestines and creating a more favorable environment for the growth of beneficial bacteria. Components of the B-vitamin group, such as vitamin B1 (thiamine) and vitamin B6 (pyridoxine), function as coenzyme in bacterial metabolic processes, thereby promoting bacterial growth. Furthermore, certain vitamins can inhibit harmful bacteria, such as *Escherichia coli*. For example, the active form of vitamin D (1,25-dihydroxyvitamin D) possesses antibacterial effects, suppressing the growth of *Escherichia coli* (69), while the antioxidant properties of vitamin E reduce the survival capacity of harmful bacteria under oxidative stress. By influencing the balance of the gut microbiota, vitamin D indirectly regulates the immune system, thereby decreasing the risk of allergic diseases, including allergic airway diseases (69, 70).

The gut microbiota can also influence the absorption and utilization of vitamins. By modulating the intestinal micro-environment, such as pH levels and redox states, the gut microbiota can affect the efficiency of vitamin absorption. For instance, SCFAs produced by certain bacteria can reduce the pH of the intestines, thereby enhancing the solubility and absorption rate of specific vitamins. Additionally, the gut microbiota can promote vitamin production and utilization by metabolizing vitamin precursors. For example, certain bacteria species can convert dietary plant sterols into vitamin D precursors, thus facilitating vitamin D synthesis. The interaction between vitamins and the gut microbiota plays a crucial role in maintaining gut health and overall health. During allergic inflammation, the antibacterial properties of vitamin D and the regulatory effects of the gut microbiota can synergistically alleviate inflammatory symptoms. Adjusting vitamin intake and modulating the composition of the gut microbiota can serve as a strategy for preventing and treating allergic diseases. For example, supplementing with vitamin D and probiotics can optimize the structure of the gut microbiota, enhance the function of the intestinal mucosal barrier, prevent allergens from entering the bloodstream, and mitigate systemic allergic reactions.

In addition to influencing the gut microbiota, vitamin metabolism plays a critical role in the body's immune system and the synthesis of specific substances. Vitamin deficiency may lead to various diseases. The vitamin D receptor (VDR) and 1α-hydroxylase are expressed in various immune cells, including T lymphocytes, B lymphocytes, DCs, neutrophils, and monocytes. This enables these cells to produce calcitriol, the active form of vitamin D3 (71). Vitamin D can suppress the production of IgE, which is associated with allergic diseases, by inhibiting the Th2 response (72). Th2 cells play a crucial role in allergic reactions, as they promote IgE generation through the secretion of IL-4 and IL-13. These processes can be inhibited by vitamin D, thereby alleviating allergic reactions (73, 74). If vitamin D reduces IgE levels against specific allergens (e.g., dust mites), it can mitigate the intensity of asthma- related allergic reactions. Vitamin D can regulate the production of various cytokines, including reducing the secretion of pro-inflammatory cytokines such as IL-5, which play an important role in allergic reactions. By inhibiting the production of these cytokines, vitamin D helps reduce allergic inflammation (75). Multiple studies have shown that IL-5 levels are positively correlated with eosinophil counts and the degree of airway inflammation in asthma patients (76). Anti-IL-5 monoclonal antibodies (such as mepolizumab) have been used to treat severe eosinophilic asthma with significant efficacy (77). Vitamin D can reduce the cytotoxic release of eosinophils, such as the release of peroxidase and necrotic factors, which can cause tissue damage during allergic inflammation (78). Overall, vitamin D is considered to have anti-inflammatory effects. Research at Chang Gung University College of Medicine in Taiwan found a significant correlation between vitamin D-related metabolites (such as 3-hydroxyisobutyrate and glutamine) and metabolites related to allergic diseases (such as succinic acid and proline) (79). This correlation is not only highly significant statistically, but also has important biological implications. For example, glutamine plays a key role in regulating immune responses and maintaining intestinal mucosal integrity (80), while succinic acid directly promotes inflammation by regulating the signaling transduction and metabolism of immune cells; meanwhile, alanine indirectly regulates inflammation by affecting the function and metabolic state of T cells. This indicates that vitamin D may affect childhood allergic airway diseases by influencing gut microbiota and immune allergic reactions (81). Studies from the Fourth Military Medical University have demonstrated that obesity-related asthma, a special phenotype of asthma, is associated with vitamin D deficiency. Obesity may result in increased storage of vitamin D in adipose tissue, thereby reducing its circulating concentrations (82, 83). The risk of asthma is significantly elevated in obese individuals, and obesity-related asthma often exhibits poor responsiveness to conventional treatments, frequently accompanied by metabolic disturbance. Rapid weight gain during childhood represents the strongest predictor for the development of asthma. Multiple clinical studies have revealed that the serum vitamin D levels in obese asthmatic patients are inversely correlated with asthma symptom severity, with lower vitamin D levels being linked to more severe symptoms. Additionally, studies have indicated that obese asthmatic patients with lower vitamin D levels also tend to exhibit reduced lung function parameters, such as forced expiratory volume in one second (FEV1) and forced vital capacity (FVC), suggesting that vitamin D may play a role in modulating lung function (82, 84, 85). Research indicates that vitamin D levels are negatively correlated with the severity of symptoms of AR (86). Vitamin D supplementation may help alleviate symptoms of these allergic diseases and improve patients' quality of life (87). Other studies suggest that supplementing vitamin D during pregnancy may reduce the risk of children developing asthma and allergic diseases (88, 89). Although existing research indicates a link between vitamin D deficiency and an increased risk of allergic diseases, the correlation is not consistent across different populations. More research is needed in populations of varying ages, genders, races, and geographic regions to clarify the relationship between vitamin D deficiency and allergic diseases in the future.

## 5 Bacteria amino acids and allergic diseases

Gut microbiota are capable of synthesizing and metabolizing a diverse array of amino acids, generating various biologically active metabolites. These metabolites play critical roles in the physiological and pathological processes of the host, including certain essential amino acids that the host is unable to synthesize autonomously (90).These amino acids are crucial for the host's energy metabolism. During amino acid metabolism, gut microbiota produce a range of metabolic products, such as SCFAs, ammonia, hydrogen sulfide, and others. These amino acids and their metabolites not only influence the survival and functionality of the gut microbiota themselves but also exert significant effects on the host's intestinal health, immune regulation, and metabolic homeostasis.

Metabolites derived from amino acid produced by the gut microbiota contribute to maintaining the balance of gut microbiota and the integrity of the intestinal mucosa. For example, fructooligosaccharides in the human intestinal tract cannot be degraded by digestive enzymes but can be fermented and utilized by the gut microbiota. Fructooligosaccharides promotes the growth of beneficial bacteria, such as *Bifidobacteria* and *Lactobacilli*, inhibits the proliferation of harmful bacteria, and enhances nutrients absorption in the intestines, thereby preserving the equilibrium of the gut microbiota (91, 92). Additionally, amino acid metabolites produced by the gut microbiota help sustain immune tolerance in the intestinal mucosa, preventing excessive immune responses. These metabolites strengthen tight connections between intestinal epithelial cells, improve the integrity of the intestinal barrier, and prevent the invasion of pathogens and harmful substances (93).

Bacteria-derived amino acids and their metabolic products can modulate the activity and function of immune cells. For instance, D-Tryptophan has been shown to decrease the production of Th2 cytokines and chemokines in both human peripheral immune cells and murine immune cells, thereby inhibiting the progression of allergic airway disease in mice (94). Additionally, tryptophan metabolities influence the balance between Th17 and Treg cells by activating AhR. Activation of AhR promotes Treg differentiation while suppressing Th17 differentiation, thus regulating immune responses and preventing allergic airway diseases such as asthma (91, 95). Furthermore, bacterial amino acids and their metabolites exhibit anti-inflammatory properties by inhibiting the production and release of pro-inflammatory factors, such as bacterial tryptophan metabolites (e.g., indole-3-propionic acid) inhibit NF-*κ*B and reduce the release of pro-inflammatory factors through activation of the PXR/TLR4 pathway, which promotes the subsidence of inflammation and accelerates the repair and regeneration of tissues (96).

The gut microbiota are capable of metabolizing amino acids and generating a variety of metabolites, among which tryptophan metabolites play a particularly critical role. Given their importance in regulating immune responses and maintaining gut microbiota, tryptophan metabolites may serve as potential therapeutic for allergic diseases. Modulating the levels of tryptophan metabolites or their metabolic pathways could potentially alleviate the symptoms and improve the prognosis of allergic diseases.

## 6 Bile acids and allergic diseases

Bile acids are primarily categorized into two types: primary bile acids and secondary bile acids. These amphiphilic molecules are derived from cholesterol, mainly synthesized in the liver, and secreted into the intestine via bile. In terms of immune regulation, bile acids modulte various physiological functions, including immune responses and the composition of the gut microbiota, by binding to specific receptors (97, 98).

Bile acids suppress allergic reactions by regulating immune cells through the activation of specific receptors. The Farnesoid X receptor (FXR) and G protein-coupled bile acid receptor (TGR5) are the primary receptors involved in this process (97). In food allergies, bile acids inhibit NF-*κ*B activation by activating FXR, thereby reducing the production of pro-inflammatory cytokines such as TNF-α, IL-6, and IL-1β. Additionally, bile acids promote the secretion of anti-inflammatory cytokines, such as IL-10, by activating TGR5, exerting anti-inflammatory effects (95, 97, 99, 100). This mechanism promotes the differentiation of Tregs, inhibites the differentiation of Th2 cells, and consequently reduces allergic reactions. Research indicates that bile acids inhibit the maturation and activation of DCs by modulating retinoic acid signaling pathways within DCs, diminishing their antigen-presenting capacity and suppressing T cells activation. This process affects the sensitization process of food allergens. Changes in the bile acid profile induced by antibiotics can enhance retinoic acid signaling in mucosal DCs, thereby promoting the production of food allergen-specific IgE and IgG1 (101). The production of allergen-specific IgG1 and IgE is a hallmark of allergic responses.

Bile acids influence the intestinal environment through interactions with the gut microbiota. They promote the growth of beneficial bacteria, such as *Bifidobacterium* and *Lactobacillus*, while inhibiting the proliferation of harmful bacteria, including *Escherichia coli*. This modulation of the gut microbiota composition helps maintain microbial balance, reduce intestinal inflammation, and further prevent systemic allergic reactions (97). Additionally, bile acids enhance the expression of tight junction proteins, such as ZO-1 and claudin, on intestinal epithelial cells by activating the FXR. This process reduces intestinal permeability and prevents the occurrence of systemic allergic inflammation symptoms (51).

Bile acids play a clinically significance role in regulating immune responses and allergic diseases. By modulating diet or administering bile acid supplements, FXR and TGR5 can be activated, thereby suppressing inflammatory responses, alleviating symptoms of allergic diseases, and providing novel therapeutics strategies and targets for the management of these conditions (99).

## 7 Conclusion

In recent years, with the rapid advancement of urbanization and changes in lifestyle, there has been a significant increase in the incidence of allergic diseases. The WHO predicts that allergic diseases associated with industrialization and Western lifestyles will double in the future, potentially due to gut microbiota imbalance. In contemporary medicine and life sciences, research into gut microbiota imbalance and its role in the prevention of allergic diseases has gained increasing attention. Investigation the interactions between the microbiome, metabolome, and host, as well as their collaborative mechanisms in maintaining intestinal homeostasis, has become a critical factor in developing more convenient and efficient treatment methods, as well as enabling early disease prevention. With the advent of high-throughput sequencing technology, we have achieved a deeper understanding of the composition and diversity of gut microbiota. The extensive application of multi-omics technologies, including metagenomics, metatranscriptomics, metaproteomics, and metabolomics, enables us to study the interactions between gut microbiota and hosts at multiple levels. These studies provide a theoretical foundation for the development of diagnostic markers and therapeutic strategies based on gut microbiota.

In the context of personalized treatment, a randomized controlled trial (*N* = 1,591 participants) demonstrated that eight out of nine probiotics could alleviate at least one clinical symptom of allergic rhinitis. Furthermore, lactate, ornithine, and six additional metabolites were identified as potential predictors of the efficacy of sublingual immunotherapy for allergic rhinitis. These metabolites regulate metabolic pathways, modulate immune system function, and mitigate symptoms of allergic rhinitis. Conversely, another double-blind randomized controlled study revealed that a probiotic mixture did not exhibit significant therapeutic effects on moderate to severe atopic dermatitis in children. Regarding the overall treatment process for allergic diseases, probiotics can alleviate allergy symptoms, reduce medication usage, and lower medical expenses. However, prolonged use may increase costs. Metabolite supplementation can enhance treatment effectiveness, decrease disease recurrence, and reduce long-term medical expenses. Nevertheless, the high cost of metabolite supplements may also lead to increased treatment expenses.

Notwithstanding these findings, there are notable limitations in the gut microbiota research. Significant inter-individual variability in gut microbiota communities limits the generalizability of research findings. Therefore, mother-infant cohort studies and cross-regional, multi-center collaborations are essential for addressing these generalizability issues. Individual genetic background, dietary habits, lifestyle, and environmental factors all influence the composition and function of the gut microbiota, adding complexity and challenges to the research. Currently, most studies can only establish correlations between gut microbiota and disease, making it difficult to determine causation. Although some intervention studies (e.g., probiotics, prebiotics, fecal microbiota transplantation) offer insights into causal relationships, these studies often suffer from small sample sizes and lack of long-term follow-up, hindering definitive conclusions. The interaction between gut microbiota and the host is a complex process influenced by multiple factors, involving systems such as the immune, metabolic, and nervous systems. Current research often focuses on single factor or pathway, which makes it challenging to comprehensively elucidate the intricate interactive mechanisms between gut microbiota and the host.

Therefore, future research should place greater emphasis on individual differences and actively pursue personalized investigations based on individual characteristics. By integrating multiple-omics datasets, incorporating individual genetic, dietary, lifestyle information, and constructing personalized gut microbiota models, a foundation for precision diagnosis and treatment can be established. Through the application of advanced experimental designs and analysis methods, such as randomized controlled trials and causal inference analyses, further exploration of the causal relationship between gut microbiota and diseases can be achieved. Simultaneously, combining animal models with clinical studies will help validate the reliability and reproducibility of these causal relationships. Based on findings regarding the relationship between gut microbiota and diseases, novel intervention strategies have been developed, including personalized probiotics formulations, prebiotics, fecal microbiota transplantation, and microbiota-derived metabolic products. Their safety and efficacy can be confirmed through rigorous clinical trials, providing new avenues for diseases prevention and treatment. Long-term follow-up studies should be conducted to monitor the dynamic changes in gut microbiotacommunities and their impact on host health and diseases progression. Through continuous monitoring and data analysis, the long-term associations between gut microbiota communities and disease development can be elucidated, offering a basis for early warning and intervention strategies. In conclusion, while significant advancements have been made in the study of gut microbiota and metabolites, numerous challenges remain. Future research should aim to uncover the specific mechanisms underlying the roles of gut microbiota and metabolomics in allergic diseases, develop personalized treatment plans, and enhance the effectiveness of disease management.

## References

[1] Marsland BJ Gollwitzer ES. Host-microorganism interactions in lung diseases. Nat Rev Immunol. (2014) 14(12):827–35. 10.1038/nri3769 25421702

[2] Zubeldia-Varela E Barker-Tejeda TC Obeso D Villaseñor A Barber D Pérez-Gordo M. Microbiome and allergy: new insights and perspectives. J Investig Allergol Clin Immunol. (2022) 32(5):327–44. 10.18176/jiaci.0852 36219547

[3] Abril AG Carrera M Sánchez-Pérez Á Villa TG. Gut microbiome proteomics in food allergies. Int J Mol Sci. (2023) 24(3):1–3. 10.3390/ijms24032234 PMC9917015 36768555

[4] Cundell AM. Microbial ecology of the human skin. Microb Ecol. (2018) 76(1):113–20. 10.1007/s00248-016-0789-6 27245597

[5] Byrd AL Belkaid Y Segre JA. The human skin microbiome. Nat Rev Microbiol. (2018) 16(3):143–55. 10.1038/nrmicro.2017.157 29332945

[6] Baquero F Saralegui C Marcos-Mencía D Ballestero L Vañó-Galván S Moreno-Arrones ÓM Epidermis as a platform for bacterial transmission. Front Immunol. (2021) 12:774018. 10.3389/fimmu.2021.774018 34925344 PMC8671829

[7] Dickson RP Erb-Downward JR Martinez FJ Huffnagle GB. The microbiome and the respiratory tract. Annu Rev Physiol. (2016) 78:481–504. 10.1146/annurev-physiol-021115-105238 26527186 PMC4751994

[8] Man WH de Steenhuijsen Piters WA Bogaert D. The microbiota of the respiratory tract: gatekeeper to respiratory health. Nat Rev Microbiol. (2017) 15(5):259–70. 10.1038/nrmicro.2017.14 28316330 PMC7097736

[9] Fassarella M Blaak EE Penders J Nauta A Smidt H Zoetendal EG. Gut microbiome stability and resilience: elucidating the response to perturbations in order to modulate gut health. Gut. (2021) 70(3):595–605. 10.1136/gutjnl-2020-321747 33051190

[10] Krautkramer KA Fan J Bäckhed F. Gut microbial metabolites as multi-kingdom intermediates. Nat Rev Microbiol. (2021) 19(2):77–94. 10.1038/s41579-020-0438-4 32968241

[11] Kim JH Kim K Kim W. Gut microbiota restoration through fecal microbiota transplantation: a new atopic dermatitis therapy. Exp Mol Med. (2021) 53(5):907–16. 10.1038/s12276-021-00627-6 34017060 PMC8178377

[12] de Vos WM Tilg H Van Hul M Cani PD. Gut microbiome and health: mechanistic insights. Gut. (2022) 71(5):1020–32. 10.1136/gutjnl-2021-326789 35105664 PMC8995832

[13] Luo Z Jin Z Tao X Wang T Wei P Zhu C Combined microbiome and metabolome analysis of gut microbiota and metabolite interactions in chronic spontaneous urticaria. Front Cell Infect Microbiol. (2022) 12:1094737. 10.3389/fcimb.2022.1094737 36710970 PMC9874702

[14] Rowland I Gibson G Heinken A Scott K Swann J Thiele I Gut microbiota functions: metabolism of nutrients and other food components. Eur J Nutr. (2018) 57(1):1–24. 10.1007/s00394-017-1445-8 28393285 PMC5847071

[15] Machate DJ Figueiredo PS Marcelino G de Cássia Avellaneda Guimarães R Hiane PA Bogo D Fatty acid diets: regulation of gut Microbiota composition and obesity and its related metabolic dysbiosis. Int J Mol Sci. (2020) 21(11):1–2. 10.3390/ijms21114093 PMC7312778 32521778

[16] Shanahan F. The gut microbiota-a clinical perspective on lessons learned. Nat Rev Gastroenterol Hepatol. (2012) 9(10):609–14. 10.1038/nrgastro.2012.145 22890109

[17] Donald K Finlay BB. Early-life interactions between the microbiota and immune system: impact on immune system development and atopic disease. Nat Rev Immunol. (2023) 23(11):735–48. 10.1038/s41577-023-00874-w 37138015

[18] George S Aguilera X Gallardo P Farfán M Lucero Y Torres JP Bacterial gut Microbiota and infections during early childhood. Front Microbiol. (2021) 12:793050. 10.3389/fmicb.2021.793050 35069488 PMC8767011

[19] Bailey MJ Naik NN Wild LE Patterson WB Alderete TL. Exposure to air pollutants and the gut microbiota: a potential link between exposure, obesity, and type 2 diabetes. Gut Microbes. (2020) 11(5):1188–202. 10.1080/19490976.2020.1749754 32347153 PMC7524284

[20] Mahmud MR Akter S Tamanna SK Mazumder L Esti IZ Banerjee S Impact of gut microbiome on skin health: gut-skin axis observed through the lenses of therapeutics and skin diseases. Gut Microbes. (2022) 14(1):2096995. 10.1080/19490976.2022.2096995 35866234 PMC9311318

[21] Arpaia N Campbell C Fan X Dikiy S van der Veeken J deRoos P Metabolites produced by commensal bacteria promote peripheral regulatory T-cell generation. Nature. (2013) 504(7480):451–5. 10.1038/nature12726 24226773 PMC3869884

[22] McAleer JP Kolls JK. Contributions of the intestinal microbiome in lung immunity. Eur J Immunol. (2018) 48(1):39–49. 10.1002/eji.201646721 28776643 PMC5762407

[23] Chen M Su Q Shi Y. Molecular mechanism of IgE-mediated Fc*ε*RI activation. Nature. (2025) 637(8045):453–60. 10.1038/s41586-024-08229-8 39442557

[24] Abdel-Aziz MI Neerincx AH Vijverberg SJ Kraneveld AD Maitland-van der Zee AH. Omics for the future in asthma. Semin Immunopathol. (2020) 42(1):111–26. 10.1007/s00281-019-00776-x 31942640

[25] Alcazar CG Paes VM Shao Y Oesser C Miltz A Lawley TD The association between early-life gut microbiota and childhood respiratory diseases: a systematic review. Lancet Microbe. (2022) 3(11):e867–e80. 10.1016/S2666-5247(22)00184-7 35988549 PMC10499762

[26] Gunaratne AW Makrides M Collins CT. Maternal prenatal and/or postnatal n-3 long chain polyunsaturated fatty acids (LCPUFA) supplementation for preventing allergies in early childhood. Cochrane Database Syst Rev. (2015) 2015(7):Cd010085. 10.1002/14651858.CD010085.pub2 26197477 PMC8783748

[27] Mohammad S Karim MR Iqbal S Lee JH Mathiyalagan R Kim YJ Atopic dermatitis: pathophysiology, microbiota, and metabolome—a comprehensive review. Microbiol Res. (2024) 281:127595. 10.1016/j.micres.2023.127595 38218095

[28] Seiti Yamada Yoshikawa F Feitosa de Lima J Notomi Sato M Álefe Leuzzi Ramos Y Aoki V Leao Orfali R. Exploring the role of staphylococcus aureus toxins in atopic dermatitis. Toxins (Basel). (2019) 11(6):1–2. 10.3390/toxins11060321 PMC6628437 31195639

[29] Darlenski R Kozyrskyj AL Fluhr JW Caraballo L. Association between barrier impairment and skin microbiota in atopic dermatitis from a global perspective: unmet needs and open questions. J Allergy Clin Immunol. (2021) 148(6):1387–93. 10.1016/j.jaci.2021.10.002 34688495

[30] Zheng X Huang F Zhao A Lei S Zhang Y Xie G Bile acid is a significant host factor shaping the gut microbiome of diet-induced obese mice. BMC Biol. (2017) 15(1):3–5. 10.1186/s12915-017-0462-7 29241453 PMC5731064

[31] Ferenc K Sokal-Dembowska A Helma K Motyka E Jarmakiewicz-Czaja S Filip R. Modulation of the gut microbiota by nutrition and its relationship to epigenetics. Int J Mol Sci. (2024) 25(2):8–10. 10.3390/ijms25021228 PMC10816208 38279228

[32] Chen Z He S Wei Y Liu Y Xu Q Lin X Fecal and serum metabolomic signatures and gut microbiota characteristics of allergic rhinitis mice model. Front Cell Infect Microbiol. (2023) 13:1150043. 10.3389/fcimb.2023.1150043. 37180443 PMC10167002

[33] Augustine T Kumar M Al Khodor S van Panhuys N. Microbial dysbiosis tunes the immune response towards allergic disease outcomes. Clin Rev Allergy Immunol. (2023) 65(1):43–71. 10.1007/s12016-022-08939-9 35648372 PMC10326151

[34] Wopereis H van Ampting MTJ Cetinyurek-Yavuz A Slump R Candy DCA Butt AM A specific synbiotic-containing amino acid-based formula restores gut microbiota in non-IgE mediated cow’s milk allergic infants: a randomized controlled trial. Clin Transl Allergy. (2019) 9:27. 10.1186/s13601-019-0267-6 31164972 PMC6543596

[35] Pujo J Petitfils C Le Faouder P Eeckhaut V Payros G Maurel S Bacteria-derived long chain fatty acid exhibits anti-inflammatory properties in colitis. Gut. (2021) 70(6):1088–97. 10.1136/gutjnl-2020-321173 32978245

[36] Cobos-Uribe C Rebuli ME. Understanding the functional role of the microbiome and metabolome in asthma. Curr Allergy Asthma Rep. (2023) 23(2):67–76. 10.1007/s11882-022-01056-9 36525159 PMC12340730

[37] Layden BT Angueira AR Brodsky M Durai V Lowe WL Jr. Short chain fatty acids and their receptors: new metabolic targets. Transl Res J Laborat Clin Med. (2013) 161(3):131–40. 10.1016/j.trsl.2012.10.007 23146568

[38] Mann ER Lam YK Uhlig HH. Short-chain fatty acids: linking diet, the microbiome and immunity. Nat Rev Immunol. (2024) 24(8):577–95. 10.1038/s41577-024-01014-8 38565643

[39] Hays KE Pfaffinger JM Ryznar R. The interplay between gut microbiota, short-chain fatty acids, and implications for host health and disease. Gut Microbes. (2024) 16(1):2393270. 10.1080/19490976.2024.2393270 39284033 PMC11407412

[40] Dong L Tang Y Wen S He Y Li F Deng Y Fecal microbiota transplantation alleviates allergic rhinitis via CD4+ T cell modulation through gut microbiota restoration. Inflammation. (2024) 47(4):1278–97. 10.1007/s10753-024-01975-x 38294580

[41] Li M van Esch B Wagenaar GTM Garssen J Folkerts G Henricks PAJ. Pro- and anti-inflammatory effects of short chain fatty acids on immune and endothelial cells. Eur J Pharmacol. (2018) 831:52–9. 10.1016/j.ejphar.2018.05.003 29750914

[42] He J Zhang P Shen L Niu L Tan Y Chen L Short-chain fatty acids and their association with signalling pathways in inflammation, glucose and lipid metabolism. Int J Mol Sci. (2020) 21(17):2–6. 10.3390/ijms21176356 PMC7503625 32887215

[43] Song XL Liang J Lin SZ Xie YW Ke CH Ao D Gut-lung axis and asthma: a historical review on mechanism and future perspective. Clin Transl Allergy. (2024) 14(5):2,5. 10.1002/clt2.12356 PMC11060082 38687096

[44] Wu R Yuan X Li X Ma N Jiang H Tang H The bile acid-activated retinoic acid response in dendritic cells is involved in food allergen sensitization. Allergy. (2022) 77(2):483–98. 10.1111/all.15039 34365653

[45] Yeh YW Xiang Z. Mouse hygiene status-A tale of two environments for mast cells and allergy. Allergology International: Official Journal of the Japanese Society of Allergology. (2024) 73(1):58–64. 10.1016/j.alit.2023.08.008 37673735

[46] Postler TS Ghosh S. Understanding the holobiont: how microbial metabolites affect human health and shape the immune system. Cell Metab. (2017) 26(1):110–30. 10.1016/j.cmet.2017.05.008 28625867 PMC5535818

[47] Kato A. Group 2 innate lymphoid cells in airway diseases. Chest. (2019) 156(1):141–9. 10.1016/j.chest.2019.04.101 31082387 PMC7118243

[48] Cong S Wang L Meng Y Cai X Zhang C Gu Y Saussurea involucrata oral liquid regulates gut microbiota and serum metabolism during alleviation of collagen-induced arthritis in rats. Phytother Res. (2023) 37(4):1242–59. 10.1002/ptr.7681 36451529

[49] Lewis G Wang B Shafiei Jahani P Hurrell BP Banie H Aleman Muench GR Dietary fiber-induced microbial short chain fatty acids suppress ILC2-dependent airway inflammation. Front Immunol. (2019) 10:10–1. 10.3389/fimmu.2019.02051 31620118 PMC6760365

[50] Zhang L Chun Y Ho H-E Arditi Z Lo T Sajja S Multiscale study of the oral and gut environments in children with high- and low-threshold peanut allergy. J Allergy Clin Immunol. (2022) 150(3):714–20.e2. 10.1016/j.jaci.2022.04.026 35550149 PMC9463091

[51] Guo H-H Han Y-X Rong X-J Shen Z Shen H-R Kong L-F Alleviation of allergic asthma by rosmarinic acid via gut-lung axis. Phytomedicine. (2024) 126:12–4. 10.1016/j.phymed.2024.155470 38417242

[52] Kan LL Li P Hon SS Lai AY Li A Wong KC Deciphering the interplay between the epithelial barrier, immune cells, and metabolic mediators in allergic disease. Int J Mol Sci. (2024) 25(13):6913. 10.3390/ijms25136913 39000023 PMC11241838

[53] van der Hee B Wells JM. Microbial regulation of host physiology by short-chain fatty acids. Trends Microbiol. (2021) 29(8):700–12. 10.1016/j.tim.2021.02.001 33674141

[54] Trompette A Gollwitzer ES Yadava K Sichelstiel AK Sprenger N Ngom-Bru C Gut microbiota metabolism of dietary fiber influences allergic airway disease and hematopoiesis. Nat Med. (2014) 20(2):159–66. 10.1038/nm.3444 24390308

[55] Parada Venegas D De la Fuente MK Landskron G González MJ Quera R Dijkstra G Short chain fatty acids (SCFAs)-mediated gut epithelial and immune regulation and its relevance for inflammatory bowel diseases. Front Immunol. (2019) 10:277. 10.3389/fimmu.2019.00277 30915065 PMC6421268

[56] Kwak MJ Kim SH Kim HH Tanpure R Kim JI Jeon BH Psychobiotics and fecal microbial transplantation for autism and attention-deficit/hyperactivity disorder: microbiome modulation and therapeutic mechanisms. Front Cell Infect Microbiol. (2023) 13:1238005. 10.3389/fcimb.2023.1238005 37554355 PMC10405178

[57] You Y-N Xing Q-Q Zhao X Ji J-J Yan H Zhou T Gu-Ben-Fang-Xiao decoction modulates lipid metabolism by activating the AMPK pathway in asthma remission. Biomed Pharmacother. (2021) 138:3–5. 10.1016/j.biopha.2021.111403 33714782

[58] Djuricic I Calder PC. Beneficial outcomes of Omega-6 and Omega-3 polyunsaturated fatty acids on human health: an update for 2021. Nutrients. (2021) 13(7):2–3. 10.3390/nu13072421 PMC8308533 34371930

[59] Magnusson J Ekström S Kull I Håkansson N Nilsson S Wickman M Polyunsaturated fatty acids in plasma at 8 years and subsequent allergic disease. J Allergy Clin Immunol. (2018) 142(2):510–6.e6. 10.1016/j.jaci.2017.09.023 29221817

[60] Miles EA Childs CE Calder PC. Long-chain polyunsaturated fatty acids (LCPUFAs) and the developing immune system: a narrative review. Nutrients. (2021) 13(1):247. 10.3390/nu13010247 33467123 PMC7830895

[61] Georgountzou A Papadopoulos NG. Postnatal innate immune development: from birth to adulthood. Front Immunol. (2017) 8. 10.3389/fimmu.2017.00957 28848557 PMC5554489

[62] Simon AK Hollander GA McMichael A. Evolution of the immune system in humans from infancy to old age. Proc Biol Sci. (2015) 282(1821):20143085. 10.1098/rspb.2014.3085 26702035 PMC4707740

[63] Hogenkamp A Ehlers A Garssen J Willemsen LEM. Allergy modulation by N-3 long chain polyunsaturated fatty acids and fat soluble nutrients of the Mediterranean diet. Front Pharmacol. (2020) 11:1244. 10.3389/fphar.2020.01244 32973501 PMC7472571

[64] Magne F Gotteland M Gauthier L Zazueta A Pesoa S Navarrete P The Firmicutes/Bacteroidetes ratio: a relevant marker of gut dysbiosis in obese patients? Nutrients. (2020) 12(5):1474. 10.3390/nu12051474 32438689 PMC7285218

[65] Stojanov S Berlec A Štrukelj B. The influence of probiotics on the Firmicutes/Bacteroidetes ratio in the treatment of obesity and inflammatory bowel disease. Microorganisms. (2020) 8(11):2–3. 10.3390/microorganisms8111715 PMC7692443 33139627

[66] Podder I Pesqué D Carrón N González Torres PI Pujol RM Giménez-Arnau AM. Gut microbial alteration in chronic spontaneous urticaria unresponsive to second generation antihistamines and its correlation with disease characteristics- a cross-sectional case-control study. Clin Transl Allergy. (2025) 15(1):e70027. 10.1002/clt2.70027 39809718 PMC11732700

[67] Hou Y Wei D Zhang Z Guo H Li S Zhang J FABP5 Controls macrophage alternative activation and allergic asthma by selectively programming long-chain unsaturated fatty acid metabolism. Cell Rep. (2022) 41(7):111668. 10.1016/j.celrep.2022.111668 36384126

[68] Pan Y Scanlon MJ Owada Y Yamamoto Y Porter CJ Nicolazzo JA. Fatty acid-binding protein 5 facilitates the blood-brain barrier transport of docosahexaenoic acid. Mol Pharm. (2015) 12(12):4375–85. 10.1021/acs.molpharmaceut.5b00580 26455443

[69] Bellerba F Muzio V Gnagnarella P Facciotti F Chiocca S Bossi P The association between vitamin D and gut microbiota: a systematic review of human studies. Nutrients. (2021) 13(10):2–3. 10.3390/nu13103378 PMC8540279 34684379

[70] Thomas RL Jiang L Adams JS Xu ZZ Shen J Janssen S Vitamin D metabolites and the gut microbiome in older men. Nat Commun. (2020) 11(1):5997. 10.1038/s41467-020-19793-8 33244003 PMC7693238

[71] Arora J Wang J Weaver V Zhang Y Cantorna MT. Novel insight into the role of the vitamin D receptor in the development and function of the immune system. J Steroid Biochem Mol Biol. (2022) 219:106084. 10.1016/j.jsbmb.2022.106084 35202799 PMC8995385

[72] Zhang P Xu Q Zhu R. Vitamin D and allergic diseases. Front Immunol. (2024) 15:1–3. 10.3389/fimmu.2024.1420883 PMC11254667 39026686

[73] Mirzakhani H Al-Garawi A Weiss ST Litonjua AA. Vitamin D and the development of allergic disease: how important is it? Clin Exp Allergy. (2015) 45(1):114–25. 10.1111/cea.12430 25307157 PMC4369152

[74] Fangal VD Kılıç A Mirzakhani H Litonjua AA Demay MB Levy BD Vitamin D exerts endogenous control over T(H)2 cell fate and immune plasticity. iScience. (2025) 28(4):112117. 10.1016/j.isci.2025.112117 40224021 PMC11987635

[75] Prietl B Treiber G Pieber T Amrein K. Vitamin D and immune function. Nutrients. (2013) 5(7):2502–21. 10.3390/nu5072502 23857223 PMC3738984

[76] Luo J Chen W Liu W Jiang S Ye Y Shrimanker R IL-5 antagonism reverses priming and activation of eosinophils in severe eosinophilic asthma. Mucosal Immunol. (2024) 17(4):524–36. 10.1016/j.mucimm.2024.03.005 38493955 PMC11649845

[77] Domvri K Tsiouprou I Bakakos P Steiropoulos P Katsoulis K Kostikas K Effect of mepolizumab in airway remodeling in patients with late-onset severe asthma with an eosinophilic phenotype. J Allergy Clin Immunol. (2025) 155(2):425–35. 10.1016/j.jaci.2024.10.024 39521278

[78] Huang W Zhang Y Li Y Ma J Li X Jiang Y Vitamin D impedes eosinophil chemotaxis via inhibiting glycolysis-induced CCL26 expression in eosinophilic chronic rhinosinusitis with nasal polyps. Cell Communication and Signaling: CCS. (2025) 23(1):104. 10.1186/s12964-025-02078-2 39985085 PMC11844113

[79] Chang YH Yeh KW Huang JL Su KW Tsai MH Hua MC Metabolomics analysis reveals molecular linkages for the impact of vitamin D on childhood allergic airway diseases. Pediatr Allergy Immunol. (2022) 33(5):e13785. 10.1111/pai.13785 35616893

[80] Cruzat V Macedo Rogero M Noel Keane K Curi R Newsholme P. Glutamine: metabolism and immune function, supplementation and clinical translation. Nutrients. (2018) 10(11):1564. 10.3390/nu10111564 30360490 PMC6266414

[81] Shore SA Cho Y. Obesity and asthma: microbiome-metabolome interactions. Am J Respir Cell Mol Biol. (2016) 54(5):609–17. 10.1165/rcmb.2016-0052PS 26949916 PMC4942201

[82] Huang J Zhou X Dong B Tan H Li Q Zhang J Obesity-related asthma and its relationship with microbiota. Front Cell Infect Microbiol. (2023) 13:1303899. 10.3389/fcimb.2023.1303899 38292857 PMC10825962

[83] Sharma V Cowan DC. Obesity, inflammation, and severe asthma: an update. Curr Allergy Asthma Rep. (2021) 21(12):46. 10.1007/s11882-021-01024-9 34921631 PMC8684548

[84] Abdo M Waschki B Kirsten AM Trinkmann F Biller H Herzmann C Persistent uncontrolled asthma: long-term impact on physical activity and body composition. J Asthma Allergy. (2021) 14:229–40. 10.2147/JAA.S299756 33737816 PMC7966302

[85] Ali GB Bui DS Lodge CJ Waidyatillake NT Perret JL Sun C Infant body mass index trajectories and asthma and lung function. J Allergy Clin Immunol. (2021) 148(3):763–70. 10.1016/j.jaci.2021.02.020 33662371

[86] Restimulia L Pawarti DR Ekorini HM. The relationship between serum vitamin D levels with allergic rhinitis incidence and total nasal symptom score in allergic rhinitis patients. Open Access Maced J Med Sci. (2018) 6(8):1405–9. 10.3889/oamjms.2018.247 30159065 PMC6108797

[87] Yepes-Nuñez JJ Brożek JL Fiocchi A Pawankar R Cuello-García C Zhang Y Vitamin D supplementation in primary allergy prevention: systematic review of randomized and non-randomized studies. Allergy. (2018) 73(1):37–49. 10.1111/all.13241 28675776

[88] Chien MC Huang CY Wang JH Shih CL Wu P. Effects of vitamin D in pregnancy on maternal and offspring health-related outcomes: an umbrella review of systematic review and meta-analyses. Nutr Diabetes. (2024) 14(1):35. 10.1038/s41387-024-00296-0 38816412 PMC11139885

[89] Weiss ST Mirzakhani H Carey VJ O'Connor GT Zeiger RS Bacharier LB Prenatal vitamin D supplementation to prevent childhood asthma: 15-year results from the vitamin D antenatal asthma reduction trial (VDAART). J Allergy Clin Immunol. (2024) 153(2):378–88. 10.1016/j.jaci.2023.10.003 37852328 PMC11740440

[90] Lin R Liu W Piao M Zhu H. A review of the relationship between the gut microbiota and amino acid metabolism. Amino Acids. (2017) 49(12):2083–90. 10.1007/s00726-017-2493-3 28932911

[91] Yan X Yan J Xiang Q Wang F Dai H Huang K Fructooligosaccharides protect against OVA-induced food allergy in mice by regulating the Th17/Treg cell balance using tryptophan metabolites. Food Funct. (2021) 12(7):3191–205. 10.1039/D0FO03371E 33735338

[92] Pereira GA Bressan J Oliveira FLP Sant’Ana HMP Pimenta AM Lopes LL Dietary folate intake is negatively associated with excess body weight in Brazilian graduates and postgraduates (CUME project). Nutrients. (2019) 11(3):518. 10.3390/nu11030518 30823410 PMC6471576

[93] Roager HM Licht TR. Microbial tryptophan catabolites in health and disease. Nat Commun. (2018) 9(1):3294. 10.1038/s41467-018-05470-4 30120222 PMC6098093

[94] Kepert I Fonseca J Müller C Milger K Hochwind K Kostric M D-tryptophan from probiotic bacteria influences the gut microbiome and allergic airway disease. J Allergy Clin Immunol. (2017) 139(5):1525–35. 10.1016/j.jaci.2016.09.003 27670239

[95] Yang W Cong Y. Gut microbiota-derived metabolites in the regulation of host immune responses and immune-related inflammatory diseases. Cell Mol Immunol. (2021) 18(4):866–77. 10.1038/s41423-021-00661-4 33707689 PMC8115644

[96] Venkatesh M Mukherjee S Wang H Li H Sun K Benechet AP Symbiotic bacterial metabolites regulate gastrointestinal barrier function via the xenobiotic sensor PXR and toll-like receptor 4. Immunity. (2014) 41(2):296–310. 10.1016/j.immuni.2014.06.014 25065623 PMC4142105

[97] Collins SL Stine JG Bisanz JE Okafor CD Patterson AD. Bile acids and the gut microbiota: metabolic interactions and impacts on disease. Nat Rev Microbiol. (2023) 21(4):236–47. 10.1038/s41579-022-00805-x 36253479 PMC12536349

[98] Ridlon JM Gaskins HR. Another renaissance for bile acid gastrointestinal microbiology. Nat Rev Gastroenterol Hepatol. (2024) 21(5):348–64. 10.1038/s41575-024-00896-2 38383804 PMC11558780

[99] Cai J Sun L Gonzalez FJ. Gut microbiota-derived bile acids in intestinal immunity, inflammation, and tumorigenesis. Cell Host Microbe. (2022) 30(3):289–300. 10.1016/j.chom.2022.02.004 35271802 PMC8923532

[100] Jia W Li Y Cheung KCP Zheng X. Bile acid signaling in the regulation of whole body metabolic and immunological homeostasis. Sci China Life Sci. (2024) 67(5):865–78. 10.1007/s11427-023-2353-0 37515688

[101] Shamji MH Valenta R Jardetzky T Verhasselt V Durham SR Würtzen PA The role of allergen-specific IgE, IgG and IgA in allergic disease. Allergy. (2021) 76(12):3627–41. 10.1111/all.14908 33999439 PMC8601105

