# Correction: Gut microbiota-metabolome crosstalk in allergic diseases: mechanistic insights and translational opportunities

**DOI:** 10.3389/falgy.2025.1669427

**Published:** 2025-09-15

**Authors:** HanBin Qin, Jiaxin Sui, Shuang Wang, Xiaojing Lv, Zile Zhang, Xinhua Lin, Xuexia Liu, Hua Zhang

**Affiliations:** 1 School of Clinical Medicine, Shandong Second Medical University, Weifang, China; 2 Department of Otorhinolaryngology, Head and Neck Surgery, Yantai Yuhuangding Hospital, Qingdao University, Yantai, China; 3 Shandong Provincial Key Laboratory of Neuroimmune Interaction and Regulation, Yantai, China; 4 Shandong Provincial Clinical Research Center for Otorhinolaryngologic Diseases, Yantai, China; 5 Yantai Key Laboratory of Otorhinolaryngologic Diseases, Yantai, China; 6 Qingdao Medical College of Qingdao University, Qingdao, China; 7 The 2nd Medical College of Binzhou Medical University, Yantai, China; 8 Shandong Stem Cell Engineering Technology Research Center, Central Laboratory, Affiliated Yantai Yuhuangding Hospital of Qingdao University, Yantai, China

**Keywords:** allergic diseases, gut microbiota, metabolome, microbiota dysbiosis, mechanisms of immunity

In the published article, there was an error. The content presented in paragraphs 5 and 6 of the Conclusion section was duplicated text present in the captions for Figures 1 and 2.

A correction has been made to **Conclusion**, Paragraphs 5 and 6. These paragraphs have have been removed.

The original article has been updated.

